# Preconditioning lung collapse: effects of early recruitment maneuvers on lung deflation dynamics during one-lung ventilation

**DOI:** 10.3389/fmed.2026.1820884

**Published:** 2026-05-19

**Authors:** Jie Zhu, Xuying Li, Wenyue Kang, Lin Wang, Guanwen Lin, Duozhi Wu, Zhihua Wang

**Affiliations:** Department of Anesthesiology, Hainan General Hospital, Hainan Affiliated Hospital of Hainan Medical University, Haikou, Hainan, China

**Keywords:** lung collapse, one-lung ventilation, pulmonary complications, recruitment maneuvers, thoracoscopic surgery

## Abstract

**Background:**

Rapid and adequate lung collapse is essential for optimal surgical exposure during video-assisted thoracoscopic surgery (VATS). Although recruitment maneuvers (RMs) are widely used to improve alveolar aeration, whether their application immediately after tracheal intubation facilitates lung collapse during one-lung ventilation remains uncertain. This randomized controlled trial evaluated the effects of immediate post-intubation RMs on lung collapse dynamics, perioperative oxygenation, and postoperative pulmonary complications.

**Methods:**

Sixty adult patients undergoing elective VATS were randomized to a recruitment maneuver group (RM, *n* = 30) or a control group (*n* = 30). The RM group received standardized recruitment maneuvers after double-lumen tube intubation, whereas the control group same standardized ventilatory strategy without recruitment maneuvers. Lung collapse quality was assessed using the Campos score after pleural opening. Time to complete lung collapse, surgeon-rated satisfaction, arterial blood gasses and postoperative pulmonary complications within 7 days were recorded (ChiCTR2300075148).

**Results:**

Compared with standardized ventilatory strategy, immediate post-intubation RMs significantly shortened the time to complete lung collapse (14.4 ± 3.3 min vs. 16.9 ± 3.7 min, *p* = 0.008). The RM group demonstrated superior early lung collapse quality, reflected by higher Campos scores within 5–15 min after pleural opening and greater surgeon-rated satisfaction. Oxygenation was higher in RM group at 15 min of one-lung ventilation. The incidence of postoperative pulmonary complications was similar between groups.

**Conclusion:**

Recruitment maneuvers performed immediately after intubation were associated with accelerated lung collapse and improved early lung collapse quality. These findings support a potential role for early recruitment maneuvers in optimizing early surgical exposure during one-lung ventilation.

**Clinical trial registration:**

https://www.chictr.org.cn/, ChiCTR2300075148.

## 1 Introduction

Video-assisted thoracoscopic surgery (VATS) has become the preferred approach for a wide range of thoracic procedures, largely because it reduces surgical trauma and facilitates enhanced postoperative recovery compared with open thoracotomy (1–3). Adequate surgical exposure during VATS, however, critically depends on rapid and complete collapse of the nondependent lung during one-lung ventilation (OLV). Delayed or incomplete lung collapse may obscure the operative field, increase technical difficulty, prolong operative time, and prompt repeated intraoperative interventions that can destabilize ventilation and oxygenation. Despite advances in lung isolation devices and ventilatory management, achieving predictable and timely lung collapse remains a persistent clinical challenge in thoracic anesthesia.

From a physiological standpoint, lung collapse during OLV is not a purely passive phenomenon but a dynamic, multiphase process (4). Immediately after pleural opening, the lung undergoes a rapid initial deflation driven primarily by elastic recoil. This is followed by a slower absorption-dependent phase, during which residual gas within distal airways and alveoli is gradually absorbed into the pulmonary circulation (4). The efficiency of this latter phase appears to be a key determinant of overall lung deflation quality. Experimental and clinical studies suggest that small airway patency and preexisting ventilation homogeneity substantially influence gas absorption, whereas airway closure, ventilation heterogeneity, and microatelectasis may trap residual gas, delay alveolar emptying, and result in incomplete or uneven lung collapse (5, 6).

Several intraoperative strategies have been proposed to accelerate lung collapse, including bronchial suction, transient ventilatory disconnection, and carbon dioxide insufflation (7–9). However, reported benefits are inconsistent, and these techniques may be limited by invasiveness, hemodynamic effects, or additional procedural complexity (7). Importantly, most of these approaches address lung collapse only after lung isolation has already been established, rather than targeting modifiable physiological conditions that precede OLV.

Recruitment maneuvers (RMs), which transiently increase transpulmonary pressure to reopen collapsed alveoli and small airways (10), are widely used as part of lung-protective ventilation strategies during general anesthesia. By restoring small airway patency and improving ventilation homogeneity, RMs have been shown to reduce atelectasis and enhance oxygenation in various perioperative and critical care settings (11). From a mechanistic perspective, these effects may also favor subsequent gas absorption during OLV and promote more uniform lung deflation. However, prior investigations have predominantly focused on RMs applied during established OLV or as rescue interventions after atelectasis has developed (12). Whether RMs performed early—immediately after tracheal intubation and before lung isolation—can precondition the lung by optimizing small airway patency and thereby facilitate subsequent lung collapse has not been systematically evaluated.

We therefore conducted a randomized controlled trial to test the hypothesis that recruitment maneuvers applied immediately after double-lumen tube intubation improve lung deflation dynamics during VATS. Specifically, we assessed the effects of early RMs on lung collapse time and quality, perioperative oxygenation, and postoperative pulmonary complications. By targeting a potentially modifiable physiological window before OLV, this study seeks to provide mechanistic insight into lung collapse dynamics and to inform a simple, reproducible anesthetic strategy that may enhance surgical conditions without adding procedural complexity.

## 2 Methods

### 2.1 Study design, ethics, and registration

This single-center, single-blind, randomized controlled trial was conducted at Hainan General Hospital (Hainan Affiliated Hospital of Hainan Medical University) between September 1, 2023 and December 31, 2023. The study protocol was approved by the Research Ethics Board of Hainan General Hospital, and written informed consent was obtained from all participants before enrollment. The trial was prospectively registered with the Chinese Clinical Trial Registry (ChiCTR2300075148; registered August 22, 2023).

### 2.2 Participants

Adult patients aged 18–65 years with a body mass index (BMI) of 18–30 kg/m^2^ and American Society of Anesthesiologists (ASA) physical status I–III, scheduled for elective video-assisted thoracoscopic surgery (VATS) requiring one-lung ventilation (OLV) with a left-sided double-lumen tube (DLT), were eligible for inclusion.

Exclusion criteria included impaired expiratory function (forced expiratory volume in 1 s [FEV1] < 70% predicted), interstitial lung disease, pleural adhesions, chronic obstructive pulmonary disease or emphysema, pneumothorax, prior thoracic surgery or chest radiotherapy, and procedures directly involving the left main bronchus (e.g., left main bronchus sleeve resection or carinal resection). Patients with severe comorbidities, including advanced hepatic dysfunction, chronic renal failure, New York Heart Association class IV heart failure, or cerebrovascular disease, were also excluded.

### 2.3 Randomization, allocation concealment, and blinding

Randomization was performed using a computer-generated random sequence (SPSS random number generator) prepared by an independent researcher. Allocation concealment was ensured using sequentially numbered, opaque, sealed envelopes, which were opened by an independent nurse immediately before anesthesia induction.

Participants and surgeons were blinded to group assignment. Because the intervention was performed intraoperatively, the attending anesthesiologist could not be blinded. Lung collapse was assessed by two experienced thoracic surgeons blinded to group allocation. Prior to the study, evaluators underwent standardized training, and a pilot assessment demonstrated substantial inter-observer agreement (Cohen’s kappa = 0.77). To minimize bias, the surgeons were not involved in the anesthetic management or the recruitment protocol.

### 2.4 Anesthesia and ventilation management

Standard intraoperative monitoring was applied according to institutional practice. Anesthesia was induced with intravenous sufentanil (0.5 μg/kg), midazolam (0.05 mg/kg), etomidate (0.3 mg/kg), and cisatracurium (0.2 mg/kg). After induction, a left-sided DLT (Hisern, 32–39 Fr, selected according to sex and height) was inserted. Correct tube position was confirmed bilaterally using fiberoptic bronchoscopy (Olympus) by an experienced anesthesiologist and reconfirmed after lateral positioning. Following intubation, both groups received two-lung ventilation (TLV) with identical ventilator settings. Detailed ventilatory parameters at predefined time points are provided in Supplementary Table S1. In brief, a lung-protective ventilation strategy was applied in both groups. During TLV, tidal volume was set at 7 mL/kg predicted body weight with a PEEP of 5 cmH₂O and FiO₂ 1.0. During OLV, tidal volume was reduced to 5 mL/kg with a PEEP of 5 cmH₂O and FiO₂ of 0.6, which was adjusted as needed to maintain adequate oxygenation. Fresh gas flow was maintained at 2 L/min, end-tidal carbon dioxide (PETCO₂) was maintained at 35–45 mmHg, and peak airway pressure was kept below 35 cmH₂O. All patients received ventilation with 100% oxygen for at least 10 min before initiation of OLV. This approach was applied uniformly in both groups to minimize potential confounding related to oxygen concentration.

The OLV was initiated with a fraction of inspired oxygen (FiO₂) of 0.6, targeting peripheral oxygen saturation (SpO₂) ≥ 92%. In cases of intraoperative hypoxemia (SpO₂ < 92%), a standardized rescue protocol was applied, including stepwise escalation of FiO₂ to 1.0, application of a recruitment maneuver if indicated, and brief return to TLV if hypoxemia persisted. Anesthesia was maintained with propofol (4–8 mg/kg/h) and remifentanil (0.1–1 μg/kg/min). Hemodynamic management followed routine clinical practice, targeting a mean arterial pressure of 70–100 mmHg and a heart rate of 50–100 beats/min. All patients underwent right internal jugular venous catheterization for routine intraoperative venous access and fluid management.

### 2.5 Intervention

Participants were allocated to one of two groups: Recruitment maneuver group (RM): Immediately after DLT intubation and confirmation of tube position, a standardized recruitment maneuver was performed in manual mode, consisting of a sustained inflation to an airway pressure of 35 cmH₂O for 15 s, with a 5-s interval between inflations, repeated 3 times (13, 14). TLV was then continued using the same ventilator settings as in the control group. Control group (C): No recruitment maneuver was performed. TLV was initiated immediately after intubation using identical ventilator settings. To minimize interference with lung collapse assessment, surgeons were instructed not to ligate the pulmonary vasculature during the first 20 min after initiation of OLV.

### 2.6 Intraoperative assessment of lung collapse

After pleural opening and visualization of the lung, thoracic surgeons assessed lung collapse at predefined time points using three measures: the Campos score (1 = very poor/no collapse; 4 = excellent/complete collapse), a lung collapse score ranging from 0 (no collapse) to 10 (complete collapse), and surgeon-rated satisfaction scored from 0 (very dissatisfied) to 10 (very satisfied) (15, 16). All assessments were performed by trained evaluators blinded to group assignment.

### 2.7 Outcomes and time points

The primary outcome was the time from pleural opening to complete lung collapse, defined as full apposition of the lung to the mediastinum without visible residual inflation (Campos score ≥4). Secondary outcomes included lung collapse scores, Campos scores at predefined time points, arterial oxygenation (PaO₂ at 15 and 30 min during OLV), intraoperative hypoxemia, operative time, surgeon-rated satisfaction, lung compliance, and postoperative pulmonary complications (PPCs). Time points were defined as follows: T0, after inhalation of 100% oxygen for 5 min; T1, immediately after intubation; T2, pleural opening; T3–T6, 5, 10, 15, and 20 min after pleural opening. Mean arterial pressure, heart rate, and SpO₂ were recorded at all time points.

### 2.8 Sample size calculation

Sample size was calculated based on the primary outcome (time to complete lung collapse), using data from a preliminary (pilot) study. The expected difference between groups and corresponding standard deviation were estimated from the pilot data. With a two-sided *α* of 0.05 and a power of 90% (*β* = 0.10), a minimum of 24 patients per group was required. Considering a dropout rate of 20%, a total of 60 patients were included.

### 2.9 Postoperative analgesia and follow-up

All patients received patient-controlled intravenous analgesia before leaving the operating room. Postoperative pulmonary complications were assessed and recorded for 7 days after surgery.

### 2.10 Definition of postoperative pulmonary complications

Postoperative pulmonary complications were defined according to the 2015 European Perioperative Clinical Outcome (EPCO) (17) definitions and were analyzed as exploratory safety outcomes.

## 3 Results

### 3.1 Participant flow and baseline characteristics

**Figure 1:**
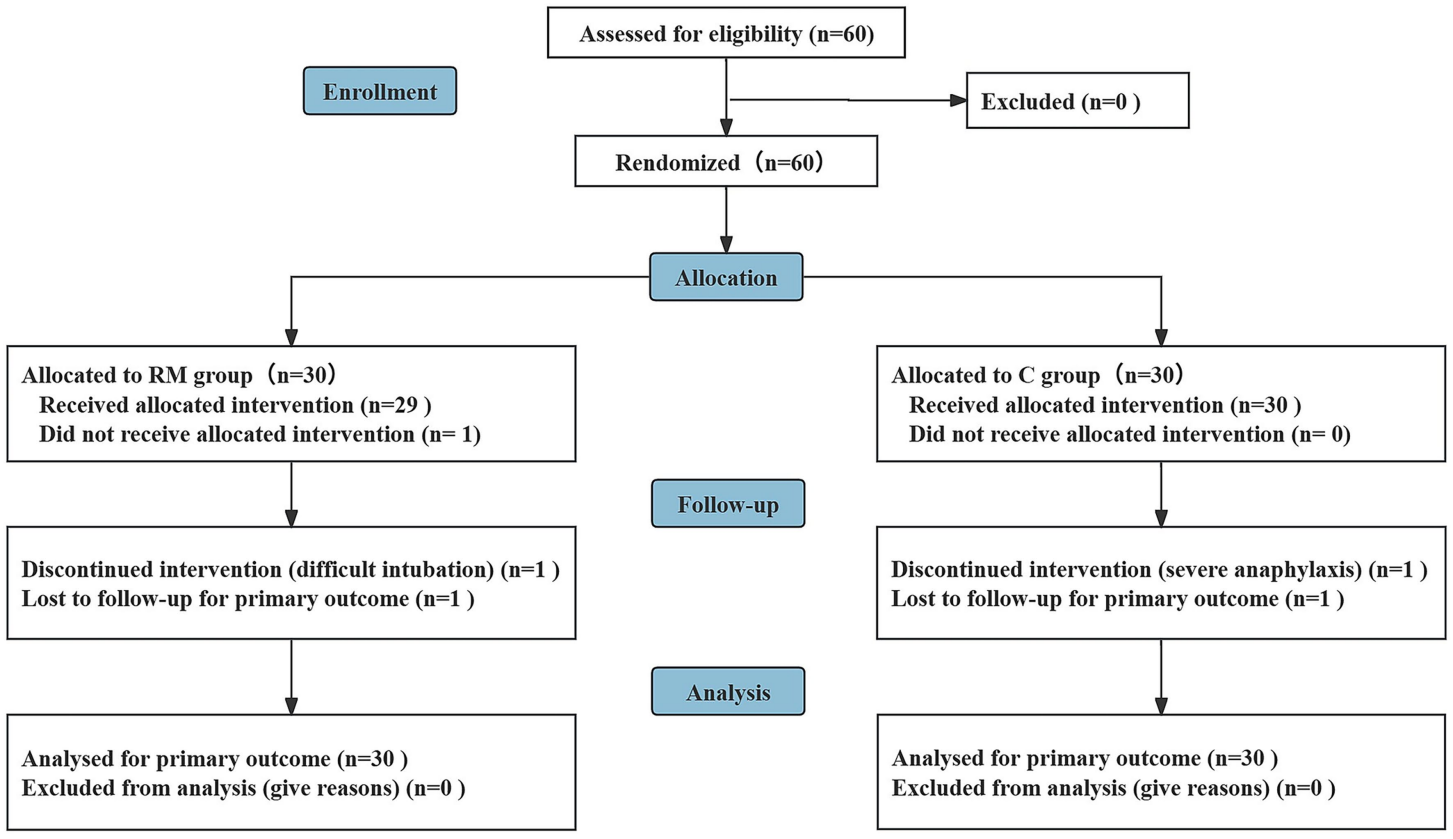
Patient flow diagram.

A total of 60 patients were enrolled and randomized. In the RM group, one patient experienced difficult intubation and one patient was lost to follow-up. In the control group, one patient developed severe intraoperative anaphylaxis and one patient was lost to follow-up. The CONSORT flow diagram is shown in Figure 1.

Baseline demographic and clinical characteristics were well balanced between groups (Table 1), with no significant differences in age, sex, BMI, pulmonary function, surgical type, comorbidities, or smoking status. Prespecified subgroup analyses by surgical type, surgical side, and baseline pulmonary function demonstrated consistent effects of the intervention on the primary outcome, with no significant interaction detected.

**Table 1:** Patient characteristics.

Variable	RM group	C group	*p*
Age (years)	49.4 ± 10.3	51.6 ± 9.6	0.373
BMI (kg/m^2^)	22.6 ± 3.1	23.2 ± 2.6	0.369
Sex (male: female)	14:16	9:21	0.222
ASA physical status (n)			0.598
I	3	1	
II	22	24	
III	5	5	
FEV^1^/FVC	78.6 ± 1.9	79.0 ± 3.0	0.567
RV/TLC	34.7 ± 4.1	36.3 ± 3.2	0.091
Side of thoracotomy (right: left)	13:17	9:21	0.333
Smoking (n)	8	5	0.349
History of lung disease (n)	1	1	1
Type of surgery (n)			0.718
Lobectomy	19	21	
Wedge resection	5	4	
Segmentectomy	4	2	
Mediastinal surgery	2	3	

Data are means ± SD. BMI, body mass index; FEV^1^, forced expiratory volume in 1 s; FEV, forced expiratory volume at 1 s; FVC, forced vital capacity (lung function test); RV, residual volume; FRC, functional residual capacity.

### 3.2 Lung collapse timing and quality

Time from pleural opening to complete lung collapse was significantly shorter in the RM group than in the control group (14.4 ± 3.3 min vs. 16.9 ± 3.7 min; mean difference −2.5 min, 95% CI − 4.13 to −0.65; *p* = 0.008). The standardized effect size indicated a moderate treatment effect (Cohen’s *d* = −0.72; Hedges’ *g* = −0.72). Sensitivity analysis using a nonparametric approach yielded consistent results (Mann–Whitney *U* = 205.00; *p* = 0.010; Hodges–Lehmann median difference −2.7 min, 95% CI − 3.5 to −1.9) (Supplementary Table S2).

**Figure 2:**
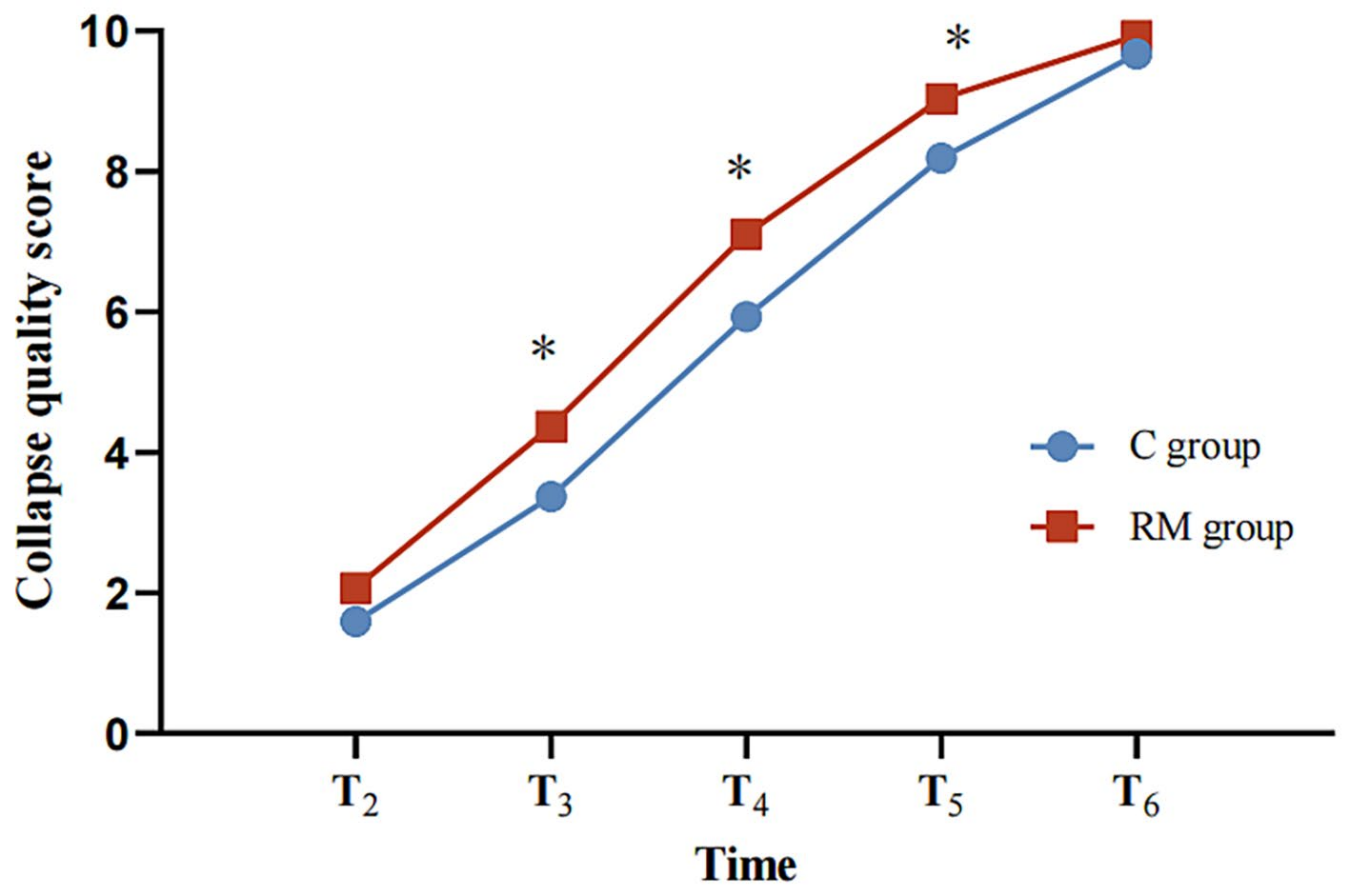
Lung collapse score chart. * indicates *p* < 0.05 vs C group.

**Figure 3:**
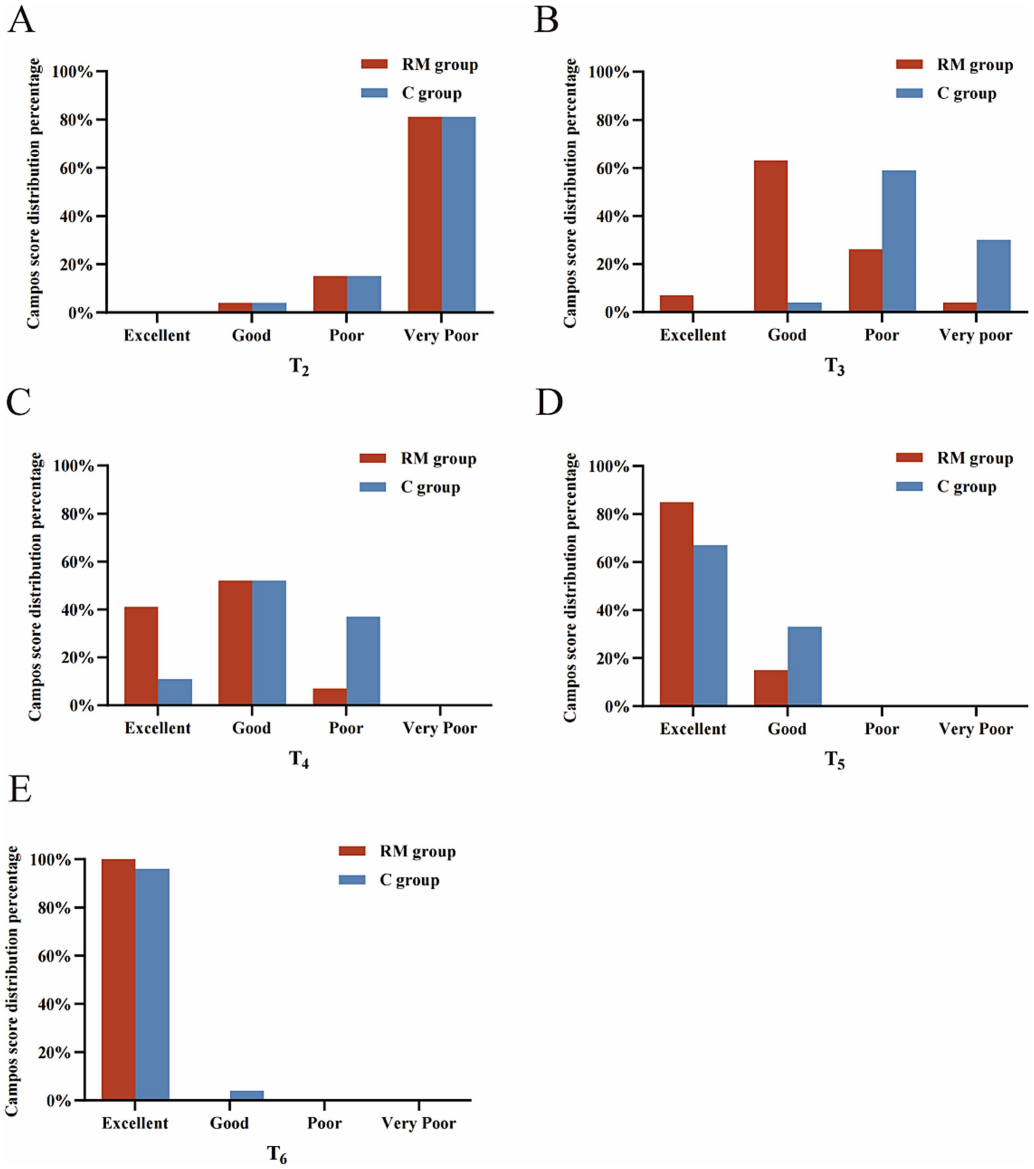
Distribution of Campos score. **(A)** The distribution of Campos score at T2. **(B)** The distribution of Campos score at T3. **(C)** The distribution of Campos score at T4. **(D)** The distribution of Campos score at T5. **(E)** The distribution of Campos score at T6.

Early lung collapse quality was superior in the RM group, with higher lung collapse scores at T3–T5 and higher Campos scores at T3 and T4 (Table 2, Supplementary Table S2 and Figure 2). At T3, a greater proportion of patients in the RM group achieved a Campos score classified as “good,” whereas at T4 and T5 a higher proportion achieved “excellent” lung collapse (Supplementary Table S2 and Figure 3). By T6, nearly all patients in both groups achieved complete lung collapse.

**Table 2:** Surgical satisfaction score.

Time point	RM group	C group	*p*
T_2_	2 (2, 3)	2(2, 3)	0.433
T_3_	5 (3, 5)^*^	3(3, 4)	0.008
T_4_	7 (6, 8)^*^	6(5, 7)	<0.001
T_5_	9 (9, 10)^*^	8(7, 10)	0.002
T_6_	10 (10, 10)	10(9, 10)	0.172

Data are median (interquartile range) as appropriate. *p < 0.05 vs. C group.

### 3.3 Oxygenation and hemodynamic variables

Hemodynamic parameters remained stable and comparable between groups throughout the study period (Supplementary Tables S3–S5). Intraoperative hypoxemia occurred in one patient in each group and was promptly corrected using the predefined rescue protocol (Supplementary Table S6). Oxygenation, expressed as the PaO₂/FiO₂ ratio, was higher in the RM group at 15 min after pleural opening (*p* < 0.05), whereas no between-group difference was observed at 30 min (Table 3).

**Table 3:** General conditions intraoperative and postoperative pulmonary complications.

Variable	RM group	C group	*p*
Hypoxemia (*n*) (%)	1 (3.3%)	1 (3.3%)	1
Operation Time (min)	92.3 ± 33.7^*^	110.2 ± 33.1	0.045
100% O_2_ ventilation time (min)	34.2 ± 7.5	36.3 ± 7.2	0.307
Oxygenation Index in 15 min	261.8 ± 120.7^*^	208.2 ± 69.9	0.044
Oxygenation Index in 30 min	196.7 ± 80.1	194.6 ± 64.5	0.904
Postoperative pulmonary complications (*n*) (%)	3 (10.0%)	2 (6.7%)	1

Data are means ± SD or median (interquartile range) as appropriate. *p < 0.05 vs. C group.

### 3.4 Operative characteristics and postoperative outcomes

Operative time was shorter in the RM group than in the control group (92.3 ± 33.7 min vs. 110.2 ± 33.1 min; *p* < 0.05). Lung compliance, airway pressure, and end-tidal carbon dioxide did not differ significantly between groups at any time point (Supplementary Table S6). Postoperative pulmonary complications occurred in 10.0% of patients in the RM group and 6.7% in the control group (risk difference 3.3, 95% CI −10.6 to 17.2%). There were no significant differences in the incidence of PPCs between groups. However, it is important to note that this study was not powered to detect changes in these exploratory safety outcomes, and these findings should be interpreted with caution. Safety outcomes, including hypoxemia, hemodynamic instability, bronchospasm, airway trauma, arrhythmias, and unplanned rescue maneuvers, were comparable between groups, and no serious adverse events were observed (Supplementary Table S7).

## 4 Discussion

In this randomized controlled trial, recruitment maneuvers applied immediately after tracheal intubation were associated with faster lung collapse and superior early lung collapse quality during video-assisted thoracoscopic surgery, without an increase in perioperative adverse events. Early oxygenation during one-lung ventilation was transiently improved, whereas postoperative pulmonary outcomes were comparable between groups. Taken together, these findings suggest that recruitment maneuvers, when applied at a specific early time point, can modulate lung collapse dynamics during thoracoscopic surgery without compromising safety.

While a reduction of approximately 2.5 min in lung collapse time is numerically modest, its clinical value lies in the optimization of the surgical workflow. In high-volume VATS centers, accelerating early deflation ensures optimal visualization immediately upon pleural opening, thereby minimizing the need for repetitive suctioning or manual lung compression. This “physiologic preconditioning” provides a more predictable and stable operative environment during the critical initial phases of hilar dissection.

Lung collapse during one-lung ventilation is increasingly understood as a dynamic and time-dependent physiological process rather than a purely passive consequence of pleural opening. While the initial phase of collapse is largely driven by elastic recoil, the subsequent absorption-dependent phase plays a central role in determining the rate and uniformity of lung deflation (18). Accumulating evidence indicates that this phase is strongly influenced by small airway patency and ventilation homogeneity before lung isolation (18). The use of 100% oxygen prior to one-lung ventilation may have facilitated lung collapse through absorption mechanisms; however, as it was applied uniformly across groups, it is unlikely to have affected the between-group comparison.

In this context, recruitment maneuvers performed immediately after intubation may act as a form of physiological preconditioning, reopening collapsed or poorly ventilated distal airways and reducing ventilation heterogeneity before the onset of one-lung ventilation (19–21). The observed acceleration in deflation dynamics suggests a potential improvement in small airway patency. However, as direct imaging such as electrical impedance tomography (EIT) was not utilized, this remains a plausible physiological hypothesis. Other factors, such as enhanced gas redistribution, reduced microatelectasis prior to one-lung ventilation and more efficient early alveolar emptying may also contribute to these findings (22). Therefore, the accelerated lung collapse and improved early collapse quality observed in this study should be interpreted as being physiologically consistent with these mechanisms, rather than as direct evidence of them.

Importantly, the observed reduction in lung collapse time primarily reflected changes in the early deflation phase after pleural opening, rather than differences in the final extent of lung collapse (23). This interpretation is supported by higher lung collapse scores during the first 5–15 min, whereas nearly all patients in both groups ultimately achieved complete lung collapse. These findings suggest that recruitment maneuvers influence the dynamics of lung deflation rather than its eventual completeness. From a clinical perspective, this distinction is relevant, as optimal surgical exposure is most critical during the early intraoperative period, when delayed or uneven lung collapse may interfere with thoracoscopic visualization and procedural efficiency. Different recruitment maneuver strategies have been described, including stepwise or incremental approaches that allow gradual pressure application. While such strategies may offer advantages in certain clinical contexts, we selected a sustained inflation approach to ensure procedural simplicity and reproducibility during routine intraoperative management.

Previous studies have explored the application of recruitment maneuvers during OLV, consistently demonstrating improvements in oxygenation and ventilation distribution (20). However, their impact on lung collapse dynamics has not been systematically evaluated. Consistent with the proposed mechanism, recruitment maneuvers were associated with improved oxygenation during the early phase of one-lung ventilation, as reflected by a higher PaO₂/FiO₂ ratio at 15 min. However, the improvement in oxygenation at 15 min was transient. This likely reflects a short-lived recruitment effect that provides a physiologic buffer during the critical transition to OLV—a period highly susceptible to hypoxemia. This effect should not be interpreted as evidence of sustained clinical benefit or improved long-term oxygenation. The transient nature of this effect aligns with prior observations that the physiological benefits of a single recruitment maneuver, such as improved ventilation distribution and increased functional residual capacity, are time limited. Notably, global lung compliance did not differ between groups, suggesting that early recruitment primarily affected regional ventilation and small airway patency rather than inducing persistent changes in overall lung mechanics.

Our findings should also be interpreted in the context of previous studies investigating strategies to improve lung collapse during thoracoscopic surgery. A range of strategies has been proposed to facilitate lung collapse during thoracoscopic surgery, reflecting the complex and multifactorial nature of lung collapse. These approaches can be broadly categorized according to their underlying physiological mechanisms. Techniques such as the use of specific inspired gas mixtures, including nitrous oxide or high concentrations of oxygen (5, 24), as well as delayed opening or preemptive one-lung ventilation (6, 8), primarily act by accelerating gas absorption from the non-ventilated lung. Similarly, brief ventilatory disconnection prior to lung isolation has been shown to enhance early deflation by initiating the absorption-dependent phase before pleural opening (25, 26). In contrast, direct mechanical interventions such as bronchial suction aim to facilitate gas evacuation (9), but their effectiveness has been variable and may be limited in certain clinical contexts. Despite these differences, the overall effect of these strategies is generally consistent, in that early intraoperative interventions may accelerate the initial phase of lung collapse and improve surgical exposure. However, the recruitment maneuver evaluated in the present study differs in both mechanism and timing. Rather than directly promoting gas removal or modifying inspired gas composition, recruitment maneuvers are applied to optimize alveolar aeration and ventilation homogeneity before lung isolation. This “preconditioning” approach targets the lung prior to the onset of one-lung ventilation, rather than attempting to modify collapse after lung isolation has been established. From this perspective, the findings of the present study extend existing literature by suggesting that early physiological optimization may influence subsequent lung deflation dynamics and improve early surgical conditions.

Postoperative pulmonary complications are a major contributor to morbidity after thoracic surgery (27). In the present study, no differences in postoperative pulmonary complications were observed between groups. However, because this outcome was not prespecified for formal hypothesis testing and was analyzed only exploratorily, the finding should be interpreted cautiously. Importantly, no safety signals or increases in adverse events were associated with recruitment maneuvers, supporting the tolerability of this approach when applied immediately after intubation.

This study was designed as a physiologic proof-of-concept. Therefore, patients with COPD or significant obstructive ventilatory defects were deliberately excluded to minimize confounding variables such as air-trapping. Consequently, our findings represent an ‘ideal-case’ scenario. While this establishes a necessary mechanistic foundation, the safety and efficacy of early recruitment maneuvers in high-risk pulmonary cohorts who may be more prone to delayed collapse, remain to be determined in future pragmatic trials. Several limitations warrant consideration. First, the follow-up period was limited to 7 days and may not have captured late-onset pulmonary complications. Second, postoperative pulmonary outcomes were not prespecified efficacy endpoints and were analyzed descriptively. Third, only a single recruitment maneuver was evaluated; whether repeated or protocolized recruitment strategies confer additional benefit remains unknown. Fourth, lung collapse assessment relied on subjective scoring, and objective measurements of regional ventilation or lung aeration were not obtained, which limits the mechanistic interpretation of the observed effects. Future studies incorporating objective imaging or ultrasound-based measures may provide further mechanistic insight. Finally, the single-center design and modest sample size may limit generalizability.

## 5 Conclusion

In conclusion, recruitment maneuvers performed immediately after tracheal intubation were associated with accelerated lung deflation, improved early lung collapse quality, and transient enhancement of oxygenation during thoracoscopic surgery, without increasing perioperative risk. By targeting modifiable physiological conditions before one-lung ventilation, this strategy may facilitate surgical exposure during the critical early phase of thoracoscopic procedures. Larger multicenter studies are warranted to confirm these findings and to identify patient populations most likely to benefit.

## Data Availability

The raw data supporting the conclusions of this article will be made available by the authors, without undue reservation.

## References

[1] Klapper J D’Amico T. VATS versus open surgery for lung cancer resection: moving toward a minimally invasive approach. J Natl Compr Cancer Netw. (2015) 13:162–4. doi: 10.6004/jnccn.2015.0023 25691607

[2] Mentzer SJ DeCamp MM Harpole DH Sugarbaker DJ. Thoracoscopy and video-assisted thoracic surgery in the treatment of lung cancer. Chest. (1995) 107:298S–301S. doi: 10.1378/chest.107.6_supplement.298s 7781410

[3] Napolitano MA Sparks AD Werba G Rosenfeld ES Antevil JL Trachiotis GD. Video-assisted Thoracoscopic surgery lung resection in United States veterans: trends and outcomes versus thoracotomy. Thorac Cardiovasc Surg. (2022) 70:346–54. doi: 10.1055/s-0041-1728707, 34044463

[4] Ng CSH He JX Rocco G. Innovations and technologies in thoracic surgery. Eur J Cardiothorac Surg. (2017) 52:203–5. doi: 10.1093/ejcts/ezx192, 28838099

[5] Ko R McRae K Darling G Waddell TK McGlade D Cheung K . The use of air in the inspired gas mixture during two-lung ventilation delays lung collapse during one-lung ventilation. Anesth Analg. (2009) 108:1092–6. doi: 10.1213/ane.0b013e318195415f, 19299766

[6] Zhang Y Yan W Fan Z Kang X Tan H Fu H . Preemptive one lung ventilation enhances lung collapse during thoracoscopic surgery: a randomized controlled trial. Thorac Cancer. (2019) 10:1448–52. doi: 10.1111/1759-7714.13091, 31115153 PMC6558447

[7] Brock H Rieger R Gabriel C Pölz W Moosbauer W Necek S. Haemodynamic changes during thoracoscopic surgery the effects of one-lung ventilation compared with carbon dioxide insufflation. Anaesthesia. (2000) 55:10–6. doi: 10.1046/j.1365-2044.2000.01123.x, 10594427

[8] Li Q Zhang X Wu J Xu M. Two-minute disconnection technique with a double-lumen tube to speed the collapse of the non-ventilated lung for one-lung ventilation in thoracoscopic surgery. BMC Anesthesiol. (2017) 17:80. doi: 10.1186/s12871-017-0371-x, 28619111 PMC5472948

[9] Quan X Yi J Huang Y Zhang X Shen L Li S. Bronchial suction does not facilitate lung collapse when using a double-lumen tube during video-assisted thoracoscopic surgery: a randomized controlled trial. J Thorac Dis. (2017) 9:5244–8. doi: 10.21037/jtd.2017.11.63, 29312732 PMC5757031

[10] Lachmann B. Open up the lung and keep the lung open. Intensive Care Med. (1992) 18:319–21. doi: 10.1007/BF01694358 1469157

[11] Tusman G Bohm SH Sipmann FS Maisch S. Lung recruitment improves the efficiency of ventilation and gas exchange during one-lung ventilation anesthesia. Anesth Analg. (2004) 98:1604–9. doi: 10.1213/01.ANE.0000068484.67655.1A, 15155312

[12] Hartland BL Newell TJ Damico N. Alveolar recruitment maneuvers under general anesthesia: a systematic review of the literature. Respir Care. (2015) 60:609–20. doi: 10.4187/respcare.03488 25425708

[13] Cakmakkaya OS Kaya G Altintas F Hayirlioglu M Ekici B. Restoration of pulmonary compliance after laparoscopic surgery using a simple alveolar recruitment maneuver. J Clin Anesth. (2009) 21:422–6. doi: 10.1016/j.jclinane.2009.08.001, 19833275

[14] Nakahira J Nakano S Minami T. Evaluation of alveolar recruitment maneuver on respiratory resistance during general anesthesia: a prospective observational study. BMC Anesthesiol. (2020) 20:264. doi: 10.1186/s12871-020-01182-9, 33069208 PMC7568405

[15] Campos J Kernstine K. A comparison of a left-sided Broncho-Cath with the torque control blocker univent and the wire-guided blocker. Anesth Analg. (2003) 96:283. doi: 10.1097/00000539-200301000-00056, 12505967

[16] Mourisse J Liesveld J Verhagen A van Rooij G van der Heide S Schuurbiers-Siebers O . Efficiency, efficacy, and safety of EZ-blocker compared with left-sided double-lumen tube for one-lung ventilation. Anesthesiology. (2013) 118:550–61. doi: 10.1097/ALN.0b013e3182834f2d, 23299364

[17] Jammer I Wickboldt N Sander M Smith A Schultz MJ Pelosi P . Standards for definitions and use of outcome measures for clinical effectiveness research in perioperative medicine: European perioperative clinical outcome (EPCO) definitions: a statement from the ESA-ESICM joint taskforce on perioperative outcome measures. Eur J Anaesthesiol. (2015) 32:88–105. doi: 10.1097/EJA.0000000000000118, 25058504

[18] Macklem P. The physiology of small airways. Am J Respir Crit Care Med. (1998) 157:S181–3. doi: 10.1164/ajrccm.157.5.rsaa-2 9606316

[19] Odor PM Bampoe S Gilhooly D Creagh-Brown B Moonesinghe SR. Perioperative interventions for prevention of postoperative pulmonary complications: systematic review and meta-analysis. BMJ. (2020) 368:m540. doi: 10.1136/bmj.m540, 32161042 PMC7190038

[20] Tusman G Böhm S Melkun F Staltari D Quinzio C Nador C . Alveolar recruitment strategy increases arterial oxygenation during one-lung ventilation. Ann Thorac Surg. (2002) 73:1204–9. doi: 10.1016/s0003-4975(01)03624-4 11996264

[21] Unzueta C Tusman G Suarez-Sipmann F Bohm S Moral V. Alveolar recruitment improves ventilation during thoracic surgery: a randomized controlled trial. Br J Anaesth. (2012) 108:517–24. doi: 10.1093/bja/aer415, 22201185

[22] Se L S M. Bench-to-bedside review: recruitment and recruiting maneuvers. Crit Care. (2005) 9:1. doi: 10.1186/cc2934, 15693985 PMC1065091

[23] Rothen H Sporre B Engberg G Wegenius G Hedenstierna G. Reexpansion of atelectasis during general anaesthesia may have a prolonged effect. Acta Anaesthesiol Scand. (1995) 39:118–25. doi: 10.1111/j.1399-6576.1995.tb05602.x, 7725873

[24] Liang C Lv Y Shi Y Cang J Miao C. The fraction of nitrous oxide in oxygen for facilitating lung collapse during one-lung ventilation with double lumen tube. BMC Anesthesiol. (2020) 20:180. doi: 10.1186/s12871-020-01102-x, 32698777 PMC7374913

[25] Germano DC Levantesi L. Lung collapse: which strategy? Minerva Anestesiol. (2023) 89:730–2. doi: 10.23736/S0375-9393.23.17465-7 37676174

[26] Somma J Couture É Pelletier S Provencher S Moreault O Lohser J . Non-ventilated lung deflation during one-lung ventilation with a double-lumen endotracheal tube: a randomized-controlled trial of occluding the non-ventilated endobronchial lumen before pleural opening. Can J Anaesth. (2021) 68:801–11. doi: 10.1007/s12630-021-01957-9, 33797018

[27] Ball L Battaglini D Pelosi P. Postoperative respiratory disorders. Curr Opin Crit Care. (2016) 22:379–85. doi: 10.1097/MCC.0000000000000312 27168252

