# Supplementary material for: Preconditioning lung collapse: effects of early recruitment maneuvers on lung deflation dynamics during one-lung ventilation
Source: Front Med (Lausanne). 2026 May 19;13:1820884. doi: 10.3389/fmed.2026.1820884 (PMC13226533; doi:10.3389/fmed.2026.1820884)
Supplement: Supplementary file 1 [file Table_1.docx]

**Table S**1 | Ventilation settings at each time point (T_0_-T_6_)

| **Time point** | **Ventilation mode** | **FiO₂** | **VT (mL/kg)** | **PEEP (cmH₂O)** |
| --- | --- | --- | --- | --- |
| T_0_ | TLV (100% O₂ for 10 min) | 1.0 | 7 | 5 |
| T_1_ | Post-intubation TLV | 1.0 | 7 | 5 |
| T_2_-T_6_ | OLV | 0.6 | 5 | 5 |

**Table S2** | Lung collapse time and quality score

| **Variable** | **RM group** | **C group** | ***p*** |
| --- | --- | --- | --- |
| Collapse time (min) | 14.4±3.3^*^ | 16.8±3.7 | 0.008 |
| Lung collapse score |  |  |  |
| T_2_ | 2(1，2) | 1(1，2) | 0.267 |
| T_3_ | 4(4，5)^*^ | 3(3，4) | <0.001 |
| T_4_ | 7(6，8)^*^ | 6(5，7) | 0.005 |
| T_5_ | 9(8，10)^*^ | 8(7，10) | 0.044 |
| T_6_ | 10(10，10) | 10(10，10) | 0.113 |
| Campos score |  |  |  |
| T_2_ | 1(1，1） | 1（1，1) | 1 |
| T_3_ | 2(2，3）^*^ | 2(1，2) | 0.002 |
| T_4_ | 3(3，4）^*^ | 3(2，3) | 0.002 |
| T_5_ | 4(3，4） | 4(4，4) | 0.224 |
| T_6_ | 4(4，4） | 4(4，4) | 0.317 |

Data are means ± SD or median (interquartile range) as appropriate. * *p* < 0.05 vs C group

**Table S3** | Comparative analysis of MAP values (mmHg)

| **Time point** | **RM group** | **C group** | ***p*** |
| --- | --- | --- | --- |
| T_0_ | 99.1±12.1 | 103.8±13.6 | 0.169 |
| T_1_ | 71.4±7.4 | 75.8±11.1 | 0.122 |
| T_2_ | 80.7±11.9 | 82.6±9.2 | 0.505 |
| T_3_ | 82.3±10.7 | 86.1±9.2 | 0.155 |
| T_4_ | 76.0±10.7 | 80.3±8.0 | 0.087 |
| T_5_ | 71.0±9.3 | 72.7±9.1 | 0.479 |
| T_6_ | 67.7±7.5 | 70.7±6.0 | 0.094 |

Data are means ± SD.

**Table S4** | Comparative analysis of HR values (bpm)

| **Time point** | **RM group** | **C group** | ***p*** |
| --- | --- | --- | --- |
| T_0_ | 78.6±13.2 | 74.4±9.9 | 0.182 |
| T_1_ | 65.3±13.7 | 62.5±7.0 | 0.345 |
| T_2_ | 65.3±10.8 | 62.9±9.7 | 0.37 |
| T_3_ | 68.9±14.8 | 63.8±9.7 | 0.126 |
| T_4_ | 72.4±16.0 | 65.7±9.7 | 0.057 |
| T_5_ | 71.7±11.6 | 65.5±8.2 | 0.220 |
| T_6_ | 67.8±11.2 | 64.2±7.7 | 0.153 |

Data are means ± SD.

**Table S5** | Comparative analysis of SpO_2_ values (%)

| **Time point** | **RM group** | **C group** | ***p*** |
| --- | --- | --- | --- |
| T_0_ | 99.3±0.9 | 98.7±0.3 | 0.120 |
| T_1_ | 99.9±0.3 | 99.8±0.5 | 0.188 |
| T_2_ | 99.8±0.5 | 99.8±0.5 | 1 |
| T_3_ | 99.4±1.3 | 99.3±1.2 | 0.834 |
| T_4_ | 97.9±2.7 | 98.4±2.0 | 0.414 |
| T_5_ | 97.6±2.4 | 97.8±2.1 | 0.770 |
| T_6_ | 97.5±2.5 | 97.4±2.5 | 0.847 |

Data are means ± SD.

**Table S6** | Respiratory parameters

| **Variable** | **RM group** | **C group** | ***p*** |
| --- | --- | --- | --- |
| Lung compliance (L/cmH_2_O) |  |  |  |
| T_1_ | 40.5±7.8 | 37.4±6.8 | 0.340 |
| T_2_ | 25.4±8.0 | 22.7±6.2 | 0.152 |
| T_3_ | 24.5±6.8 | 23.2±5.3 | 0.507 |
| T_4_ | 25.0±7.4 | 23.6±5.6 | 0.497 |
| T_5_ | 24.6±6.4 | 23.5±5.8 | 0.578 |
| T_6_ | 24.5±6.9 | 23.8±5.4 | 0.775 |
| P_ET_CO_2_ (mmHg) |  |  |  |
| T_1_ | 33.2±3.3 | 34.9±3.5 | 0.058 |
| T_2_ | 33.8±3.5 | 34.3±3.0 | 0.525 |
| T_3_ | 35.1±3.4 | 35.7±2.8 | 0.481 |
| T_4_ | 35.9±3.2 | 36.4±2.7 | 0.507 |
| T_5_ | 34.0±4.1 | 35.5±2.7 | 0.116 |
| T_6_ | 34.1±3.3 | 34.7±2.6 | 0.450 |
| Airway pressure (cmH_2_O) |  |  |  |
| T_1_ | 17.4±2.4 | 17.0±2.3 | 0.478 |
| T_2_ | 20.3±3.6 | 20.8±3.3 | 0.595 |
| T_3_ | 20.3±3.9 | 20.6±3.1 | 0.795 |
| T_4_ | 20.0±4.0 | 20.6±2.9 | 0.547 |
| T_5_ | 20.5±4.3 | 20.5±2.7 | 0.971 |
| T_6_ | 19.9±3.8 | 20.3±2.8 | 0.579 |

Data are means ± SD. P_ET_CO_2_: end-expiratory carbon dioxide

**Table S7** | Safety assessment.

| **Event** | **Number** | **Absolute Risk (RM)** | **Absolute Risk (C)** | **Risk Difference (RD)** | **95% CI ( lower, upper )** |
| --- | --- | --- | --- | --- | --- |
| Hypoxemia | 2 | 0.033 | 0.033 | 0 | (-0.101, 0.101) |
| Unplanned Rescue Maneuvers | 1 | 0 | 0.033 | 0.33 | (-0.108, 0.034) |
| Other safety incidents | 0 | 0 | 0 | 0 | 0 |
